# Comparison of pharmacokinetics and drug release in tissues after transarterial chemoembolization with doxorubicin using diverse lipiodol emulsions and CalliSpheres Beads in rabbit livers

**DOI:** 10.1080/10717544.2017.1344336

**Published:** 2017-06-29

**Authors:** Shuisheng Zhang, Can Huang, Zhengzheng Li, Yongjie Yang, Tingting Bao, Haibo Chen, Yinghua Zou, Li Song

**Affiliations:** a Department of Interventional Radiology and Vascular Surgery, Peking University First Hospital, Beijing, China;; b Department of Radiology, Beijing Chaoyang Hospital, Capital Medical University, Beijing, China;; c The State Key Laboratory of Natural and Biomimetic Drugs, School of Pharmaceutical Sciences, Peking University, Beijing, China;; d Department of Cardiology, Second Affiliated Hospital, College of Medicine, Zhejiang University, Hangzhou, China

**Keywords:** Pharmacokinetics, rabbit liver, drug-eluting beads, CalliSpheres^®^ Beads, doxorubicin

## Abstract

CalliSpheres^®^ Beads (CB) is the first drug-eluting bead (DEB) product in China. Our aim was to compare the effect on the pharmacokinetics of doxorubicin (DOX) and its local concentration between lipiodol emulsions and CB in the process of TACE in rabbit livers. Twenty-five rabbits were distributed into two groups; Group 1 received lipiodol emulsions with DOX, and Group 2 received CB loaded with DOX (CBDOX). DOX was measured in the peripheral blood at different times after treatment. Livers were sampled at 1 week and 1 month for Group 2 after embolization. DOX concentration and distribution were measured in the liver. The administration of DOX by TACE with CBDOX resulted in peripheral blood DOX concentrations of 39.85 ± 13.86 ng/mL at 5 min, with a gradual decrease to 6.89 ± 1.62 ng/mL at 24 h, after treatment. Plasma concentration of DOX after chemoembolization with lipiodol was 225.91 ± 64.88 ng/mL at 5 min and decreased with time by 24 h to 5.06 ± 0.48 ng/mL. In CBDOX group, the drug impregnated an area as far as 200 μm from the bead edge. The tissue concentration of doxorubicin (*tissC_DOX_*) ranged from 40.27 μg/mL to 245.70 μg/mL at 1 week and from 5.64 μg/mL to 28.09 μg/mL at 1 month. Plasma concentrations of DOX resulting from CBDOX embolization were significantly lower than that for cTACE. CB could deliver relatively high concentrations of DOX to an area as far as 200 μm from the bead edge for at least 1 month.

## Introduction

Transcatheter hepatic arterial chemoembolization (TACE) is one of the most commonly used treatments for unresectable liver tumors (Oliveri et al., 2012; Fu et al., 2015; Ha et al., 2016; Li et al., 2016). TACE is commonly used with chemotherapy agents, such as epirubicin and doxorubicin (DOX) (Llovet et al., 2002; Tawada et al., 2015). DOX has a broad spectrum of anticancer activity. It has positive charges, which could make the drug easily loadable to carriers (Lewis, 2009), and the spectral characteristics of DOX are relatively clear. These features make DOX one of the best test drugs for animal studies.

Drug-eluting beads (DEB), which can be used as carriers for various antineoplastic agents in TACE, were developed in the mid-2000s (Namur et al., 2010). Doxorubicin-eluting beads (DEBDOX) have recently been studied *in vitro* and *in vivo*. *In vitro*, DEBDOX can provide a controlled release of DOX, with a half-value period greater than 2 months (Lewis et al., 2006; Gonzalez et al., 2008). *In vivo*, DOX can be detected in the liver tissue 2 weeks after embolization in rabbit livers with DEBDOX (Hong et al., 2005). DEBs have proven advantages over conventional TACE (cTACE) in both pharmacokinetic parameters in animal experiments (Hong et al., 2006; Gentil et al., 2014) and effects in clinical trials (Zou et al., 2016).

However, DEB products have not been widely used in China. CalliSpheres^®^ Beads (CB) is the first DEB product developed in China. Previous studies on CB focused on the toxicology (Guan et al., 2016), and no pharmacokinetic research was carried out to determine the advantages of the first products developed in China over traditional TACE. Several issues should be considered and examined. First, the tissue concentration of doxorubicin (*tissC_DOX_*) surrounding the beads at different times after embolization of CB loaded with DOX (DEBDOX) should be directly determined. Second, we should also compare the pharmacokinetics between CBDOX embolization and cTACE simultaneously. To address these problems, it is rather challenging to develop quantitative methods with sufficient spatial resolution to separate *tissC_DOX_* surrounding the beads. The method of tissue homogenization is not suitable for detecting *tissC_DOX_* surrounding the beads because it is difficult to exclude all beads loaded with drugs and the *tissC_DOX_* surrounding the beads varies with the distance to beads and other factors. However, fluorescence microspectroscopy can detect DOX distribution quantitatively, by using the unique spectral features of DOX (Namur et al., 2009).

In the present study, rabbits were used as the animal model. Rabbits are relatively inexpensive and easy to raise. The hepatic arteries of rabbits are suitable for interventional procedures. We compared the plasma pharmacokinetics between CBDOX embolization and cTACE in healthy rabbit livers by using liquid chromatography tandem mass spectrometry (LC–MS/MS). Simultaneously, we detected *tissC_DOX_* surrounding the beads in rabbit liver embolized with 100–300-μm CBDEB (smallest sizes available) via fluorescence microspectroscopy at two time points: 1 week and 1 month after embolization.

## Materials and methods

### Materials

Doxorubicin hydrochloride (Meilune Biological Technology Co., Ltd., Dalian, China) was reconstituted with sterile water to obtain solutions of different concentrations.

CalliSpheres^®^ Beads (Jiangsu Hengrui Medicine Co. Ltd., Jiangsu, China) measuring 100–300 μm were used as the carrier. CB at a dose of 0.1 mL was added to the DOX solution at a dose of 0.5 mL with a concentration of 8 mg/mL. Then, the mixture was kept for 2 h in a shaker at room temperature of 23–28 °C until the solution became relatively light in color. Finally, CBDOX at a fixed dose of 40 mg DOX/mL beads was well prepared. Lipiodol (Guerbet Laboratories Ltd., Paris, France) and polyvinyl alcohol (PVA) (Cook, Inc., Bloomington, IN) were also used.

Rat tail tendon collagen type I (Solarbio Science & Technology Co., Ltd., Beijing, China) at a concentration of 5 mg collagen per mL was mixed with agarase (SunShine Biotechnology Co., Ltd., Madrid, Spain) at a concentration of 0.01 g/mL at a ratio of 1:2 to produce phantoms.

### Animals and groups

This study was approved by the Ethics Committee of the Peking University First Hospital.

In this study, 25 male New Zealand rabbits aged approximately 4 months age and weighing 3.5–4.0 kg were used. All animals were bred in the animal breeding facility of the Peking University First Hospital. Animals had free access to standard pellet diets and water during the experimental period. All protocols conformed to the Regulations for the Administration of Affairs Concerning Experimental Rabbits, formulated by the Ministry of Science and Technology of China. All 25 rabbits were grouped into two groups, eight rabbits in Group 1 and 17 rabbits in Group 2. Group 1 was a control group of cTACE by lipiodol and PVA. Group 2 received CBDOX, at a dose of 0.1 mL, loaded with 4 mg DOX, and PVA and were sacrificed to obtain liver tissue 1 week (five rabbits) and 1 month (five rabbits) after embolization.

For the cTACE group, DOX solution (0.5 mL) with a concentration of 8 mg/mL and lipiodol (0.3 mL) were reconstituted within a 2 mL syringe with mixture of contrast medium and saline at a ratio of 2:1. For the CBDOX group, CBDOX (0.1 mL) was reconstituted within a 2 mL syringe with the same mixture as above. The final dose of DOX for each group was 4 mg.

### Transcatheter hepatic arterial chemoembolization

Embolization of the hepatic artery with CBDOX or lipiodol was performed in all rabbit livers. TACE was performed with digital subtraction arteriography (DSA) and fluoroscopic guidance (DSA; GE cop., Conneciticut).

All animals were anesthetized before interventional treatment through the administration of an intravenous injection of 35 mg/kg pentobarbital sodium at a concentration of 2% (Dolethal; Vetoquinol, Lure Cedex, France).

After well anesthetized, an incision was made on the rabbit to expose the right femoral artery, and then a 4-F sheath was placed into the exposed artery. Through the sheath, a 4-F catheter, usually a Cobra (Cook, Inc., Bloomington, IN), was placed into the common hepatic artery to perform celiac angiography to identify the hepatic arterial anatomy. After that, a thinner catheter, a 2.7-F microcatheter (Cook, Inc., Bloomington, IN), was used when getting to common hepatic artery. When the microcatheter was advanced into the left hepatic lobe, the embolic material was manually administered slowly under fluoroscopic monitoring carefully to avoid reflux of the embolic material into non-target vessels. We used PVA for supplement till left hepatic artery disappeared totally in the post-treatment angiography. After the completion of embolization, all catheters and sheaths were removed and the wound was sewed up.

### Sample collection

We collected serum samples 5 min, 10 min, 30 min, 1 h, 3 h, 6 h, 12 h, and 24 h after treatment for Group 1 and Group 2. At least 1 mL blood sample was collected from every rabbit though marginal ear veins at every time point. All the serum samples were processed for LC–MS/MS analysis.

Necropsy was performed 1 week and 1 month after embolization respectively for the two groups, and liver tissue was collected. Samples were taken from any noticeable area beside necrosis section. Samples were immediately embedded in medium for cryosection after freezing in liquid nitrogen, and were cut in 10-μm-thick sections with a microtome.

### LC–MS/MS analysis

To measure the DOX concentration in the serum, blank serum samples (190 μL) were spiked with DOX standard solution (10 μL) to make the standard curve in serum, which ranged from 5 ng/mL to 600 ng/mL.

Daunorubicin (Meilune Biological Technology Co., Ltd., Dalian, China) in methyl alcohol at a concentration of 2 μg/mL and dose of 10 μL, as the internal standard solution, were added to all of the standards and samples. The precipitation of protein was done according to the established protocols for LC–MS/MS (Hronek & Reed, 2015; Tao et al., 2015).

High-performance liquid chromatography (HPLC) was carried out using a Dionex UltiMate 3000 RSLC Nano (Thermo Fisher Scientific Inc., Waltham, MA). A XBridge C18 column (2.5 μm, 2.1 × 50 mm column; Waters Corporation, Dublin, Ireland) was used, with the flow rate 0.4 mL/min. The mobile phase consisted of A (acetonitrile [AcCN]) and B (0.1% formic acid, 99.9% water) at a ratio of 79:21. The injection volume of the standards or samples was 20 μL.

Mass spectrometry was carried out on an API 4000 QTRAP LC-MS/MS System (Applied Biosystems/MDS Sciex, Concord, Ontario, Canada). The lower limit of quantity (LOQ) of LC–MS/MS for DOX was 5 ng/mL.

### Microspectrofluorimetry, quantification, and calibration

All liver sample sections were assessed with a microspectrofluorimeter (Jobin Yvon HR800/Horiba, Lille, France) coupled to an argon ion laser at *λ* = 477 nm excitation (Spectra Physics, Evry, France). By using microspectrofluorimetry, DOX concentration in the liver tissue surrounding the beads was detected. Fluorescence emission spectra were detected every 10 μm from the surface of the bead linearly to a distance of 200 μm with an objective 40× magnification.

The fluorescence spectra obtained by the microspectrofluorimeter from a tissue was a compounded spectra, including the intrinsic tissue and the drug. Each contribution has unique spectral shape and the intensity of fluorescence is proportional to the concentration of material. For this study, the contributions of DOX and liver tissue were separated by direct least squares (LabSpec 4.10, Jobin Yvon/Horiba, Lille, France).

Phantoms made from collagen type I and agarase loaded with different concentrations of DOX from 5 μg/mL to 1000 μg/mL were used to performed to calibration. The contribution of DOX was calculated and plotted against the concentration of DOX to make standard calibration curve. And the standard calibration curve was linear in the concentration range of 5 ng/mL to 1000 ng/mL, with the determination coefficient *R*^2^ being 0.990.

The lower LOQ of microspectrofluorimetry for DOX in tissue was 5 μg/mL.

### Pharmacokinetics study

All the plasma concentration data in both groups were processed using PKSolver (version 2.0; China Pharmaceutical University, Nanjing, China). A non-compartment model was used, and the intravenous bolus injection procedure was selected. The maximum plasma concentration (*C*_max_), area under the curve (AUC), half-time (*T*_1/2_), and mean residence time (MRT) were calculated and compared.

### Statistical analysis

A *t*-test was used to compare the serum or tissue concentration of DOX between different groups. Independent samples test was used to compare pharmacokinetic parameters of serum DOX. Statistical analyses were performed using IBM SPSS Statistics package (version 20; IBM, Chicago, IL). The level of significance was defined as *p* < .05.

## Results

### Transcatheter hepatic arterial chemoembolization

**Figure 1.**
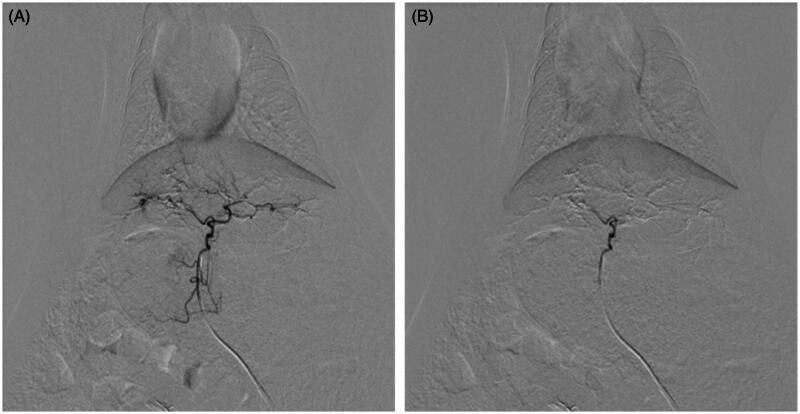
Arterial angiogram of a rabbit liver (A) before embolization and (B) immediately after embolization with CBDOX and PVA. The hepatic artery trunk was occluded with CBDOX and PVA after embolization.

TACE were performed successfully in 23 rabbits (92%) by the same operators, with TACE of one rabbit being unsuccessful in each group. No obvious reflux was seen. The endpoints of the procedure were completion of injection of embolic material, for Group 1 lipiodol emulsions loaded with DOX and for Group 2 CBDOX, and vanishment of left hepatic artery in the post-treatment angiography though PVA embolization for supplement (Figure 1).

Two rabbits (28.6%) and six rabbits (35.7%) died in Group 1 and Group 2, respectively, after embolization. The possible main causes of death were embolization procedure and toxicity of chemotherapy.

### *In vivo* pharmacokinetics of doxorubicin

**Figure 2.**
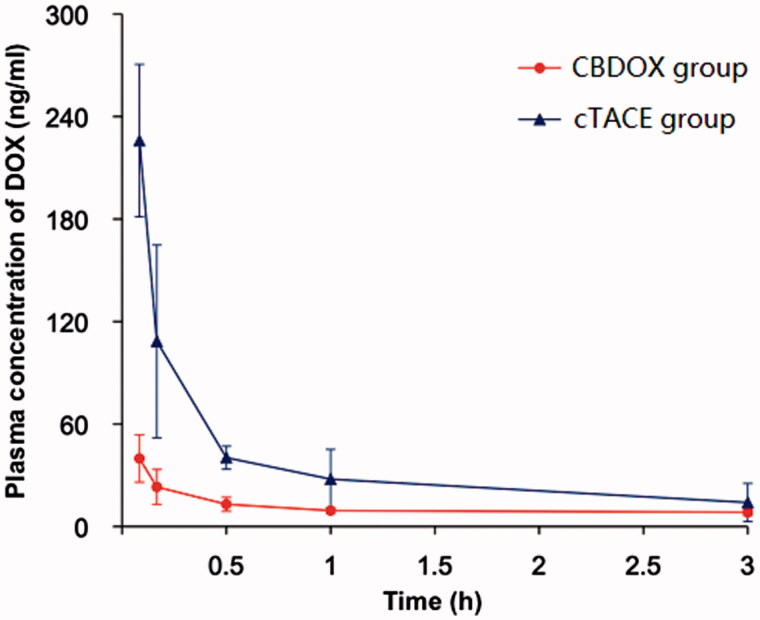
The plasma concentration curves of doxorubicin after liver artery embolization in two groups (*n* = 5). Symbols: (filled triangle) cTACE group; (filled circle) CBDOX group.

All serum samples were examined for DOX 5 min, 10 min, 30 min, 1 h, 3 h, 6 h, 12 h, and 24 h after DOX dosing, by using LC–MS/MS method. The concentration of DOX in the serum decreased over time after transarterial chemoembolization (Figure 2).

The administration of DOX by TACE with CBDOX resulted in DOX concentrations of 39.85 ± 13.86 ng/mL, 23.16 ± 10.29 ng/mL, 13.06 ± 4.08 ng/mL, 9.33 ± 1.81 ng/mL, 8.38 ± 2.01 ng/mL, 7.85 ± 2.23 ng/mL, 7.25 ± 1.46 ng/mL, and 6.89 ± 1.62 ng/mL in the peripheral blood at 5 min, 10 min, 30 min, 1 h, 3 h, 6 h, 12 h, and 24 h after treatment. While the plasma concentration of DOX after chemoembolization with lipiodol to rabbit was 225.91 ± 64.88 ng/mL, 108.40 ± 17.54 ng/mL, 40.31 ± 10.78 ng/mL, 27.78 ± 3.25 ng/mL, 14.07 ± 2.54 ng/mL, 9.26 ± 2.02 ng/mL, 6.21 ± 0.51 ng/mL, and 5.06 ± 0.48 ng/mL, respectively.

The pharmacokinetic parameters of DOX based on noncompartmental pharmacokinetic analyses are shown in Table 1. The mean *C*_max_ of DOX in the serum in cTACE group is 225.91 ± 64.88 ng/mL, significantly greater than that in CBDOX group. Also, the AUC_(0–_*_t_*_)_ in cTACE group and CBDOX group was 275.94 ± 27.45 mg/L*h and 191.18 ± 40.36 mg/L*h, with a significant difference. The *T*_1/2_ and MRT_(0–_*_t_*_)_ in cTACE group were shorter than that in CBDOX group but it is not significantly different.

**Table 1.** Pharmacokinetic parameters based on noncompartmental pharmacokinetic analyses for embolization with CBDOX or cTACE of doxorubicin solution to rabbit livers (*n* = 5).

Parameters	Units	TACE with CBDOX	cTACE
*C*_max_	ng/mL	39.85 ± 13.86	225.91 ± 64.88^#^
AUC_(0–_*_*t*_*_)_	mg/L*h	191.18 ± 40.36	275.94 ± 27.45^#^
MRT_(0–_*_*t*_*_)_	h	157.78 ± 130.86	26.58 ± 12.45
*T*_1/2_	h	109.84 ± 90.88	23.17 ± 10.43

*C*_max_: maximum plasma concentration; AUC: area under the curve; *T*_1/2_: half-time; MRT: mean residence time.

# *p*<.01.

### DOX concentration in tissue

Animals were sacrificed and liver samples were collected 1 week and 1 month after the operation in Group 2, and the DOX concentration around CB was determined by microspectrofluorimetry. A total of 10 DOX profiles were recorded: five profiles for 1 week, 5 for 1 month.

**Figure 3.**
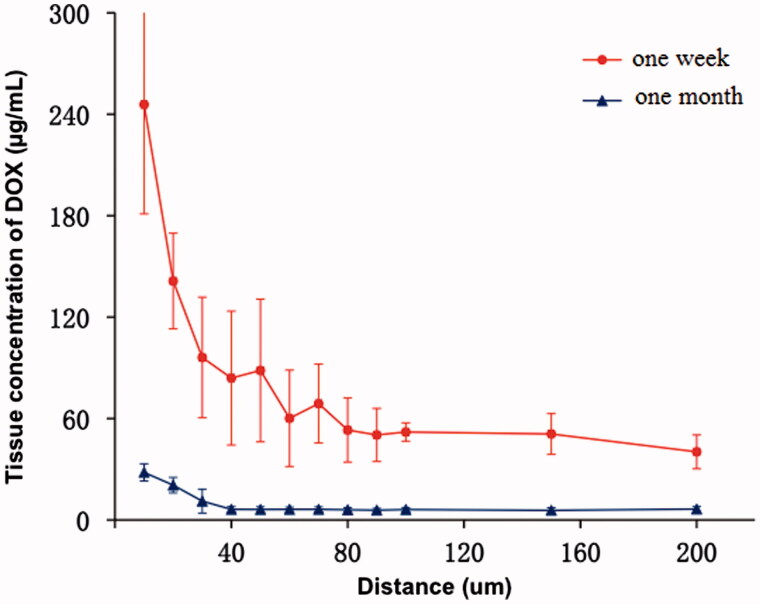
The tissue concentration curves of doxorubicin after liver artery embolization with CBDOX to rabbits (*n* = 5). Symbols: (filled triangle) 1 month group; (filled circle) 1 week group.

In the rabbit liver 1 week after embolization, *tissC_DOX_* decreased with the distance to the edge of bead, from 245.70 ± 64.66 μg/mL at the surface to 40.27 ± 10.01 μg/mL at a distance of 200 μm. The mean *tissC_DOX_* was 47.69 ± 10.41 μg/mL in the field between 100 μm and 200 μm from the beads surface. At 1 month after embolization with CBDOX, *tissC_DOX_* decreased from 28.09 ± 5.10 to 6.39 ± 1.47 μg/mL with the distance to the DEB (Figure 3). The mean *tissC_DOX_* in the field between 100 μm and 200 μm from the beads was 6.05 ± 1.33 μg/mL and was significantly lower than after 1 week (*p* < .001).

Besides, we failed to detect DOX in the liver section 1 week or 1 month after cTACE.

## Discussion

The aims of TACE are to block the tumor vasculature and increase the concentration of antitumor drugs. The efficacy of TACE depends mostly on the difference in the blood supply between normal liver and tumors (Namur et al., 2011). Unlike the normal liver, liver tumors, such as hepatocellular carcinoma (HCC), mainly rely on the hepatic artery for nutrient and oxygen supply (Breedis & Young, 1954).

TACE is commonly used with chemotherapy agents, such as epirubicin and DOX (Llovet et al., 2002; Tawada et al., 2015). In the present study, we choose DOX as our test drug. According to previous reports (Hong et al., 2006; Baumgarten et al., 2012; Gentil et al., 2014; ElSohly et al., 2015; Gholamrezanezhad et al., 2016; Meng et al., 2016), the dose of DOX used for rabbits ranged from 0.6 to 3 mg/kg; we choose a relatively small dose (1 mg/kg) because of the following two considerations: first, for the detection of DOX in tissues and serum, larger doses of DOX are more suitable. Second, for the safety and welfare of animals, smaller doses of DOX are more suitable. If we used more DOX, there would be more carriers, both of which would cause more deaths. We used the results from previous experiments to determine the optimal dose of 1 mg/kg.

Traditionally, the antitumor drug is mixed with lipiodol and injected into the hepatic artery with or without other embolic materials, such as gelatin sponge and PVA, to achieve embolization and chemotherapy. Lipiodol, which is also used as a contrast agent, acts both as an embolic agent by accumulating in tumors and as a carrier of the antitumor drug (Llovet et al., 2002; Deng et al., 2011). The injected drug mixture of ethiodized oil, antineoplastic agent, contrast agent and saline which is used in cTACE, varies considerably in nature and stability, and fails to show a controlled release pattern (Tzeng et al., 2008). The results of the present study showed a relatively high concentration of DOX in plasma within a short time after the operation, whereas we failed to detect DOX in the liver section 1 week after cTACE. This result is consistent with the experiment conducted by Gupta et al. (2011); in their report, the DOX concentration in the plasma was relatively high within 3 h after the operation, and the DOX in the liver decreased to minimal levels within three days. Another experiment, conducted by Hong et al. (2006), showed a similar result, where the concentration of DOX in tissues in the cTACE group on day 7 was very low, but not zero, compared to that of the DEB group. The different results may be caused by different animal models and different DOX doses. We used 4 mg DOX for normal rabbits (about 4 kg); Gupta et al. (2011) used 2 mg DOX for rabbit models of liver cancer (about 3 kg), whereas Hong et al. (2006) used 11.5 mg DOX for rabbit models of liver cancer (about 4 kg). The lipiodol could not be retained in the normal liver but could be retained in the tumor. Therefore, in our rabbit livers, we failed to detect DOX in the livers in the cTACE group.

Contrary to ethiodized oil, DEB has several advantages, including a controlled level of occlusion and release of the antitumor drug. The aim of TACE with DEBDOX was to provide prolonged and sustained drug delivery and a high diffusion of DOX into the liver tissue surrounding the beads.

CalliSpheres^®^ Beads is the first DEB product made in China. It is a type of ion-exchange bead with some negatively charged functional groups. These negatively charged functional groups are responsible for the loading of many positively charged drugs, such as DOX (Lewis, 2009). The present study compared the effects of two different carriers, lipiodol and CB, on the DOX concentration in serum. The administration of DOX via cTACE by using lipiodol resulted in an initial concentration of 225.9 ng/mL, which decreased to 5.06 ng/mL 24 h after treatment. On the contrary, embolization with CBDOX resulted in a relatively lower concentration. The local delivery of DOX using CBDOX embolization could help eliminate the systemic toxicities associated with cTACE, transarterial infusion or intravenous infusion (Chatziioannou et al., 2013). The results of the plasma concentrations of the two groups concur with previously published reports (Hong et al., 2006; Gentil et al., 2014) on the plasma pharmacokinetic profiles of DEBs and cTACE. In Hong’s report (Hong et al., 2006), the plasma concentration in the DEB group was lower than that in the cTACE group. Gentil et al. (2014) showed similar results, although they used a different volume ratio (lipiodol to DOX solution) of a lipiodol emulsion. The reasons for this advantage of DEB or CB over cTACE are as follows: the oil water emulsion in the cTACE is not a stable mixture, so the DOX was released rapidly. However, the combination between the beads and DOX is a combination of negative-positive charges, which is more stable. Therefore, the release of DOX in the CB group was slower. Another reason is that the beads could be occluded from the vessels for relatively longer times. However, the lipiodol could flow to other areas for some time. Therefore, the DOX in the CB group would possible be retained in the liver.

In the present study, DOX was detected in the tissue surrounding the beads 1 week and 1 month after embolization, showing that drug delivery by CB might be maintained for a period of at least 1 month after embolization. Average DOX concentrations of 50 and 6 μg/mL were measured in the liver 1 week and 1 month after TACE with CBDOX. *In vitro*, the release of DOX by DEBDOX could last for several weeks (Gonzalez et al., 2008); *in vivo*, it is suggested that DEBDOX could release chemotherapeutic drugs for a period of time longer than 2 weeks after the operation (Hong et al., 2006). Namur et al. showed that the retained DOX in DEB at 1 month could reach 57% and at 3 months 11% (Namur et al., 2010). The results are similar to those of previous analyses owing to the sustained-release of DEBs and CBs. These results also correspond to the DOX tissue concentration.

The key advantage of the present study was the distribution of the tissue concentration of DOX, with limited reports in previous studies. DOX is detected in the liver tissue around the beads, indicating that CBDOX could deliver DOX to the surrounding tissue. The present study demonstrated that DOX diffuses in the tissue radially around the beads, and the DOX concentration decreased with the distance to the beads. Each bead could deliver the drug to an area of at least 200 μm from the bead surface after TACE. A rapid decrease of drug concentration was observed beside the surface, because of the presence of a barrier (Yuan et al., 2004), and the drug tissue distribution profile around the beads showed a more gradual decrease further from the beads (Weinberg et al., 2007). Inflammatory reaction and fibrotic encapsulation are characteristic features of the foreign body reaction to implants, which have been described in detail in different organs for non-loaded embolics (Lankelma et al., 1999; Laurent et al., 2004). Two studies on breast cancer showed that an impairment of DOX diffusion in tissue was found after the i.v. injection of DOX (Lankelma et al., 1999; Primeau et al., 2005). DOX diffusion in solid tumors was limited by many factors, such as the blood compartment and clearance from plasma (Jain, 2012). However, the bead acted as a continuous and prolonged source of DOX into the tissue. A prolonged exposure of tumor cells to the chemotherapeutic agent could increase the effects of the drugs (Zheng et al., 2001).

The present study has several limitations, which should not be ignored. The major limitation is that this study, which was performed on healthy rabbits, does not represent conditions in patients with a tumor. Another limitation is that the number of animals we used was relatively small. Other drawbacks of our experimental design include that hepatic parenchymal damage in the two groups was not evaluated. To thoroughly investigate the advantage of CB in PK and to overcome the limitations, PK and drug release in tissue studies in a large number of rabbits with VX2 liver tumors are necessary. Furthermore, relative clinical trials comparing the two methods are necessary.

## Conclusions

In conclusion, the plasma concentrations of DOX result from CBDOX embolization are significantly lower than that for cTACE. DOX concentration in the tissue can last for a period of at least 1 month and DOX can diffuse over a distance of 200 μm from the surface of beads.
