# Correction to: Zhang etal., Comparison of pharmacokinetics and drug release in tissues after transarterial chemoembolization with doxorubicin using diverse lipiodol emulsions and CalliSpheres Beads in rabbit livers

**DOI:** 10.1080/10717544.2017.1352062

**Published:** 2017-07-10

Zhang S, Huang C, Li Z, Yang Y, Bao T, Chen H, Zou Y and Song L. (2017). Comparison of pharmacokinetics and drug release in tissues after transarterial chemoembolization with doxorubicin using diverse lipiodol emulsions and CalliSpheres Beads in rabbit livers. *Drug Delivery*. VOL. 24, NO. 1, pp. 1011–1017. https://doi.org/10.1080/10717544.2017.1344336.

When the above article was first published online, asterisk symbol (*) for the authors Shuisheng Zhang and Can Huang was missing which states that both the authors have contributed equally to this work. This has now been corrected in both the print and online versions.

